# Pragmatic information of aesthetic appraisal

**DOI:** 10.1007/s11571-025-10225-1

**Published:** 2025-02-06

**Authors:** Peter beim Graben

**Affiliations:** Bernstein Center for Computational Neuroscience, Berlin, Germany

**Keywords:** Empirical aesthetics, Neuroaesthetics, Dynamic semantics, Information theory, Predictive processing, Pragmatics

## Abstract

A phenomenological model for aesthetic appraisal is proposed in terms of pragmatic information for a dynamic update semantics over belief states of an aesthetic appreciator. The model qualitatively correlates with aesthetic pleasure ratings in an experimental study on cadential effects in Western tonal music, conducted by Cheung et al. (Curr Biol 29(23):4084–4092.e4, 2019). Finally, related computational and neurodynamical accounts are discussed.

## Introduction

Aesthetics is, according to Alexander Gottlieb Baumgarten (1714–1762), the science of beauty (Baumgarten 1750). Putting it more scientifically, aesthetics is the science of critical judgement of the beautiful (Nake 1974). Therefore, aesthetics is also the science of critical judgement of the ugly as the opposite of the beautiful (Stangneth 2019). In these latter senses, aesthetics is part of aesthesiology as the science of perception in general, and of the reception of art in particular (Consoli 2020; de Cruys et al. 2024; Leder et al. 2004; Tedesco 2024), thus overlapping with psychology and the human cognitive neurosciences (Fechner 1876; Frascaroli et al. 2024; Pearce et al. 2016). For the judgment of an object or event as being beautiful, abstract features, such as regularity, symmetry or orderliness are often invoked (Frascaroli et al. 2024; Weyl 1980). Formal principles such as the *golden ratio* as one specific trait appear in all kinds of natural and artistic beauty, ranging from flowers to galaxies in nature and from ancient cultures to contemporary architecture, painting, sculpture, music, dance and drama (Atlas 2003; Frascaroli et al. 2024; Leder and Pelowski 2024; Livio 2002; Power and Shannon 2019).

Also the pioneers of experimental psychology and psychophysics, Gustav Theodor Fechner (1801–1887) and Wilhelm Wundt (1832–1920) were acutely interested in aesthetic judgements. Fechner, e.g., in his *Vorschule der Ästhetik* (literally: *Primary School on Aesthetics*), had conducted the probably very first experiments on empirical aesthetics: Subjects were asked to select the most “beautiful” and the “ugliest” quadrangle from a selection of ten simultaneously presented ones with equal areas but with different aspect ratios, ranging from squares (1 : 1) to rectangles with 2 : 5, also including the golden ratio ($\varphi \approx 21:34 \approx 0.6176$) (Fechner 1876, p. 190). His results indicated that the rectangle with the golden ratio was experienced as the most beautiful stimulus (Fechner 1876, p. 195), although he conceded that the aesthetic value of the golden ratio could have been somewhat overestimated (Fechner 1876, p. 192). Yet, later attempts to replicate Fechner’s results have revealed some methodological flaws in his studies (Livio 2002, Ch. 7).

**Fig. 1:**
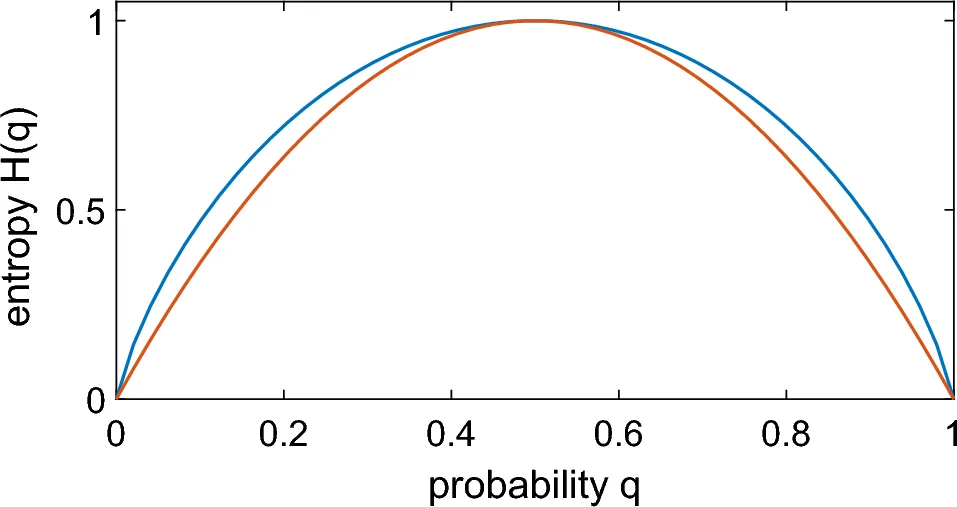
Comparison of Fermi–Dirac entropy *H*(*q*) with logistic map *L*(*q*) against probability parameter *q* of a binary distribution. Blue: Fermi–Dirac entropy (14); red: logistic map (18) (Color figure online)

Plotting the results of (Fechner 1876, p. 195) graphically, entails a kind of bell-shaped, or “inverse U-shaped” response curve that already appeared in Wundt’s *Grundzüge der Physiologischen Psychologie* (*Principles of Physiological Psychology*) as emotional strength depending on stimulus intensity (Wundt 1874, p. 432). This characteristic curve (Wundt 1874, Fig. 97); also compare with Fig. 1 below for illustration) was dubbed the “Wundt curve” of “hedonic value” by Berlyne (1970), where

[...] the abscissa is taken to represent “arousal potential,” a term meant to cover all the stimulus properties that tend to raise arousal, including novelty and complexity. Positive hedonic value reaches a maximum with moderate arousal potential (stimulation producing a moderate arousal increment) and then, as arousal potential increases further, hedonic value takes on lower and lower positive values and finally becomes negative. (Berlyne 1970, p. 284)

Here, maximal “hedonic value” corresponds with the aesthetic appreciation of the beautiful.

The Wundt curve of aesthetic appraisal has, more or less, been confirmed in numerous studies on empirical aesthetics. To mention only a few of them: McMullen and Arnold (1976) presented pairs of rhythmic musical sequences to their subjects. These were asked to choose the most preferred or most interesting sequences. As one result they reported that the beginning of a response curve displays an inverse U-shape in dependence on redundancy in rhythmic sequences. In a more recent study, Marin and Leder (2013) proposed a cross-domain approach, acknowledging the multidimensional nature of complexity. In four experiments, they employed pictures of affective scenes, representational paintings, and music excerpts. Stimuli were pre-selected to vary in emotional content and complexity. Ratings of familiarity, complexity, pleasantness and arousal were obtained for each set of stimuli. Subsequent statistical analyses revealed positive correlations between subjective complexity and arousal. Later, Güçlütürk et al. (2016) collected ratings of liking and perceived complexity for digitally generated images. Additionally, they also calculated complexity measures for each image. As a result, they reported an inverse U-curve relationship between aesthetic appreciation and stimulus complexity. In a large corpus study, Sigaki et al. (2018) and Perc (2020) conducted quantitative analyses of almost 140,000 paintings, covering nearly a millennium of art history. Based on the local spatial patterns in the images, they estimated entropy and complexity measures of each painting. These measures represented the amount of visual order of artworks, locally reflecting qualitative categories proposed by art historians. Interestingly, the dependence of statistical complexity on entropy exhibited another inverse U-curve relationship. Finally, Agres et al. (2017) investigated the connection between harmonic musical structure and aesthetic appraisal. In a psychological rating experiment listeners assessed their enjoyment for computer-generated anthems with varying levels of harmonic repetition and complexity. These ratings were further explored using computational approaches, based on information theory and on musicological tonal tension measures (Fram 2019; Huron 2006; You et al. 2023). Their results also provided evidence for a Wundt-type relationship between complexity and aesthetic enjoyment.

**Fig. 2:**
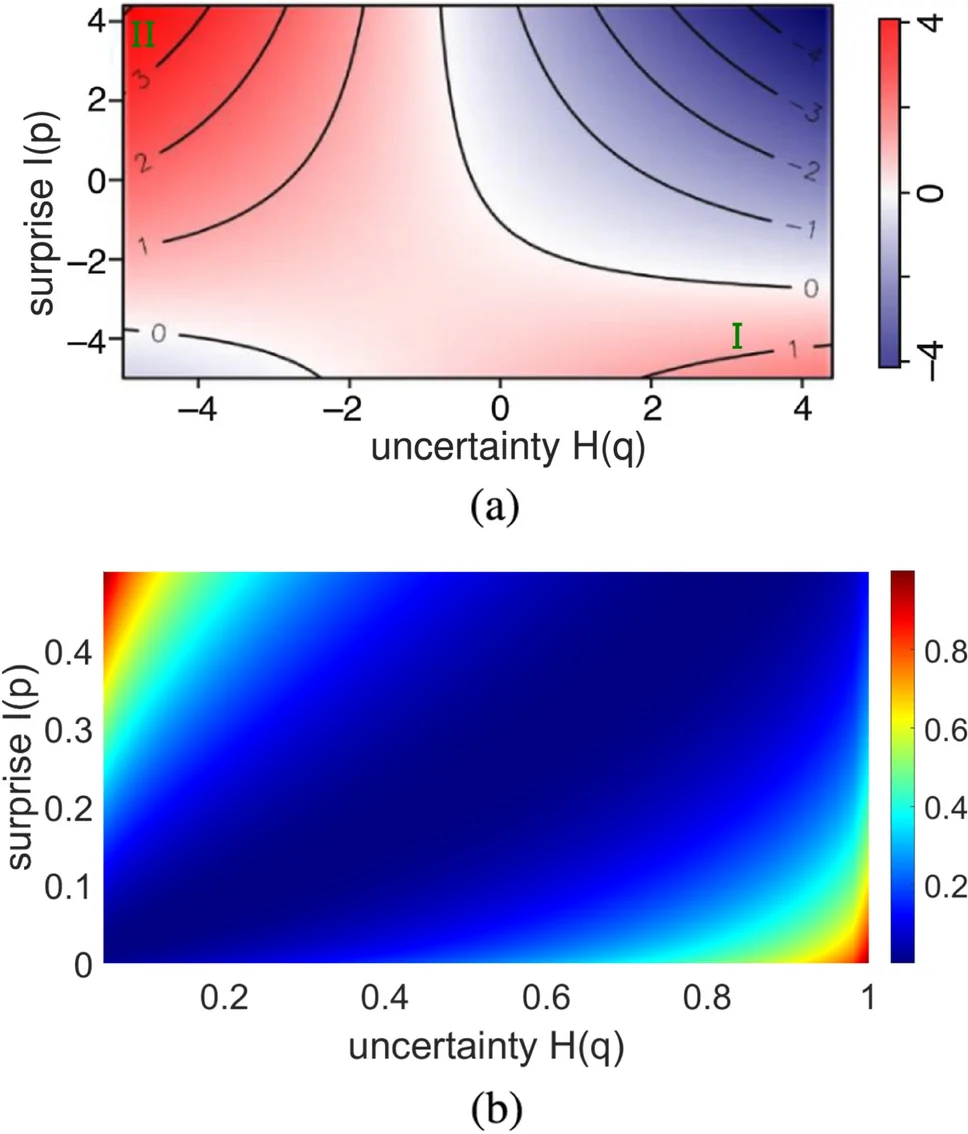
Comparison of aesthetic pleasure rating P(I‖H) from the experimental study of Cheung et al. (2019) with pragmatic information model S(I(p)‖H(q)). **a** Normalized *z*-scores of aesthetic appreciation in dependence of context uncertainty *H*(*q*) (abscissa) and stimulus surprise *I*(*p*) (ordinate). Indicated are regions (I) and (II) as discussed in the text. Reprinted after slight modifications from Cheung et al. (2019, Fig. 2(b)) with permission from Elsevier. **b** Pragmatic information (20) for probabilistic context updating dynamics (4) under a chordal operator $\textbf{A}$

Clearly, judgements of the beautiful and the reception of art works are complex neurodynamical and mental processes that require multivariate and multidimensional methodology (Consoli 2020), which was also substantially recognized in the experiments by Cheung et al. (2019, 2024) on the appreciation of musical beauty. Their results are partly reproduced in Fig. 2a below. Specifically, Cheung et al. (2019) demonstrated that pleasure varies nonlinearly as a function of two independent variables: context uncertainty and stimulus surprisal. Taking Western tonal harmony as a model of musical structure, they trained machine-learning models to mathematically quantify the uncertainty and surprise of about 80,000 chords in pop songs. They reported that chords were experienced as most pleasurable in two distinguished cases: On the one hand for highly uncertain contexts, but less surprising closure (Fig. 2a: region I). On the other hand for contexts with low uncertainty but highly surprising closure (Fig. 2a: region II). Moreover, the pleasure rating exhibited a nonlinear saddle surface whose cross section along the main diagonal of Fig. 2a displayed the characteristic inverse U-curve as well. In an additional neuroimaging study, Cheung et al. (2019) reported that activity in the amygdala, hippocampus, and auditory cortex reflected the observed interaction, while the nucleus accumbens only accounted for uncertainty. It is the aim of the present study, to discuss those results in the light of a phenomenological model of aesthetic reception.

As already mentioned, many of the studies reviewed above, employed methods from information theory (Friston et al. 2006; Moles 1966; Nake 1974, 2012; Shannon and Weaver 1949; Völz 1988), complexity science (Badii and Politi 1997; Gernert 2006), and machine learning (Pearce 2018; Russell and Norvig 2010; Schmidhuber 2010; Sun et al. 2011) which are also suitable for a deeper theoretical understanding and philosophical reflection of aesthetic judgements. An early account was given by Immanuel Kant (1724–1804) in his *Kritik der Urteilskraft* (*Critique of the Power of Judgement*), where he wrote:

[...] the judgement of taste must rest on a mere sensation of the reciprocal activity of the Imagination in its freedom and the Understanding with its conformity to law. It must therefore rest on a feeling, which makes us judge the object by the purposiveness of the representation (by which an object is given) in respect of the furtherance of the cognitive faculty in its free play. (Kant 1790/1914, p. 161, §35)

For Kant, a “judgement of taste” (i.e., an aesthetic judgement) involves two mental faculties: “Understanding” as the “faculty of rules” (Kant 1787/1999, B171) and “Imagination” as the “faculty of intuitions” (Kant 1790/1914, p. 161, §35) that are engaged in a free, harmonious play, enjoying the recipient’s mind upon the beautiful. Thus, the faculty of “Understanding” always tries to classify a beautiful object or event according to some rules of regularity, principles of symmetry, or laws of orderliness. Contrastingly, the faculty of “Imagination” permanently attempts to generate novel aspects, creative insights and fantastic associations of the given object or event (de Cruys et al. 2024; beim Graben 2024).

These characteristics of aesthetic judgements were also emphasized in the endeavor of information-theoretical aesthetics (Moles 1966; Nake 1974, 2012; Völz 1988). According to Moles (1966), complete regularity and order are experienced as *banality*, while irregular creativity and *originality* appear as randomness—both extremes not exhibiting any hedonic value. Applied to music reception, Meyer (1956) likewise observed that if

[...] the stimuli comprising the series cannot be perceived as being similar in any respect whatsoever, then they will fail to cohere, to form a group or unit, and will be perceived as separate, isolated, and discrete sounds, “signifying nothing.” Since contrast and comparison can exist only where there is similarity or equality of some sort, the mental impression created by such a series will be one of dispersion, not disparity; of diffusion, not divergence; of novelty, not variety. [...] Complete similarity, proximity, and equality of stimulation, on the other hand, will create an undifferentiated homogeneity out of which no relationships can arise because there are no separable, individual identities to be contrasted, compared, or otherwise related. There will be coexistence and constancy, but not connection; uniformity and union, but not unity. In short, both total segregation and total uniformity will produce sensation, but neither will be apprehended as pattern or shape. (Meyer 1956, p. 158)

Here again, the dichotomy between “total segregation and total uniformity” corresponds to the distinction between “novelty”, or “originality” (Moles 1966), on the one hand, and “similarity”, or “banality” (Moles 1966), on the other hand.

Another important framework for computational and empirical aesthetics, combining information theory and cognitive neurodynamics, is the predictive processing (PP) account (Frascaroli et al. 2024; Friston et al. 2006; Koelsch et al. 2019; de Cruys et al. 2024; Constant et al. 2024; Kesner 2024; Leder and Pelowski 2024; Miller et al. 2024; Pepperell 2024) that is closely related to the free energy principle (FEP) (Friston et al. 2006). Free energy is an important concept of equilibrium statistical mechanics that has been metaphorically adopted to dynamical systems theory and machine learning technology. In the context of predictive processing, the FEP states that adaptive systems are obliged to minimize expected prediction error, or, in other words, surprisal. According to Huron’s imagination - tension - prediction - reaction - appraisal (ITPTRA) theory (Huron 2006, p. 21), successful prediction is mostly rewarded by emotional pleasure:

Confirmation of expected outcomes generally induces a positive emotional response even when the expected outcome is bad. (Huron 2006, p. 13)

By contrast,

The phenomenon of ‘surprise’ represents a failure of expectation. From a biological perspective, surprise is always a bad thing. Even when the surprising outcome turns out to be good, failing to anticipate the outcome means that the brain has failed to provide useful information about possible futures. [...] If an animal is to be prepared for the future, the best surprise is no surprise. (Huron 2006, p. 21)

Therefore, there are “three strong emotions that are closely linked to surprise: laughter, awe, and frisson” (Huron 2006, p. 25) that may serve for adaptive behavior and subsequent survival. Thus, also the predictive processing account deals with confirmation and banality, i.e., predictability, on the one hand, and with surprising novelty and originality on the other hand. Note that also Kant speculated about the aesthetic roles of laughter for the appreciation of beauty (Kant 1790/1914, p. 222, §54) and of awe for the appraisal of the sublime (Kant 1790/1914, p. 136, §29), respectively.

It is the aim of the present study to present an alternative information-theoretical formalization of the banality/originality dichotomy in terms of *pragmatic information* (Gernert 2006; beim Graben 2006; Kornwachs and von Lucadou 1982; von Weizsäcker and von Weizsäcker 1972) as a phenomenological model of aesthetic appraisal (Menninghaus et al. 2019; Blutner 2024).

## Pragmatic information

In his own contribution to the foundation of information theory, Weaver described three levels of the communication problem “by which one mind may affect another” (Shannon and Weaver 1949, p. 3), namely

- Level A. How accurately can the symbols of communication be transmitted? (The technical problem.)
- Level B. How precisely do the transmitted symbols convey the desired meaning? (The semantic problem.)
- Level C. How effectively does the received meaning affect conduct in the desired way? (The effectiveness problem.) (Shannon and Weaver 1949, p. 4).

These levels correspond roughly with the dimensions of semiotics (Morris 1938). The “technical problem” with noisy communication channels can be addressed by redundant codes, introducing correlations between the symbols of the messages, i.e., by *syntax*. The “semantic problem” refers to correlations between the transmitted symbols and their intended meanings (*semantics*). Finally, the “effectiveness problem” relates to *pragmatics*, namely the correlations between the symbols and their impact upon the message users.

Shannon’s information measure *H*, which is formally related to Boltzmann’s entropy in statistical physics and thermodynamics, only accounts for the “technical problem” of efficient communication (Shannon and Weaver 1949). A tentative measure of semantic information, or informativity *I*, was proposed by Bar-Hillel and Carnap (1953). Finally, von Weizsäcker and von Weizsäcker (1972) suggested three desiderata to be fulfilled by any reasonable measure of pragmatic information *S*:

- (*i*) Pragmatic information *S* assesses the impact of a message upon the mental states of a cognitive agent.
- (*ii*) Pragmatic information *S* should vanish in the limits of novelty (originality) *N* and confirmation (banality) *C*.
- (*iii*) Pragmatic information *S* presumably requires a non-classical but rather quantum-like description in terms of incompatible mental operators.

Obviously, condition (*ii*) could be easily satisfied by defining pragmatic information as a product of suitable measures for novelty and confirmation: S=N×C, such that S=0 for either N=0 or C=0. This idea was originally suggested by Kornwachs and von Lucadou (1982).

In Shannon’s information theory (Shannon and Weaver 1949), a discrete information source is given by an *n*-dimensional probability space $(\mathscr {X}, \textbf{p})$ endowed with a probability distribution $\textbf{p} \in [0, 1]^n$, such that $p_i$ ($0 \le p_i \le 1$) is the probability of the atomic event $E_i \in \mathscr {X}$. The discrete set $\mathscr {X}$ is considered as a *repertoire* with $p_i$ being the probability that the event $E_i$ was selected (Nake 1974, 2012).

The information content, or *informativity* (Bar-Hillel and Carnap 1953), of an event $E_i$ is defined as

1 $$\begin{aligned} I(p_i) = - \,\textrm{ld}\,p_i . \end{aligned}$$

The base of the logarithm is usually chosen as two, such that $\,\textrm{ld}\,\equiv \log _2$ yields information measured in “bits”. Equation (1) indicates that the smaller the probability $p_i$, the larger the informativity $I(p_i)$, which is therefore a measure of *surprise* for the event $E_i$ (Agres et al. 2018; Hale 2016). Clearly, informativity *I* can also be regarded as a measure of novelty or originality *N* (von Weizsäcker and von Weizsäcker 1972).

The averaged information over the entire distribution entails the Shannon *entropy*

2 $$\begin{aligned} H(\textbf{p}) = \sum _i p_i \, I(p_i) = - \sum _i p_i \, \,\textrm{ld}\,p_i , \end{aligned}$$

where the natural convention $p_i \,\textrm{ld}\,p_i = 0$ if $p_i = 0$ applies (Shannon and Weaver 1949). Note that the contribution of a single event $E_i \in \mathscr {X}$ to the entropy of the information source,

3 $$\begin{aligned} S(p_i) = p_i \, I(p_i) = - p_i \, \,\textrm{ld}\,p_i = N(p_i) \times C(p_i) , \end{aligned}$$

could be regarded as a measure of pragmatic information of $E_i$ in the sense of Kornwachs and von Lucadou (1982), for $N(p_i) = I(p_i)$ assesses novelty, while $C(p_i) = p_i$ obviously reflects confirmation. This quantity has been introduced as the *conspicuity* (*Auffälligkeit*) of the event $E_i$ in the framework of information-theoretic aesthetics (Nake 1974, 2012; Völz 1988). Further notice that the function (3) assumes its local maximum at site $p_i^* = 1/\textrm{e}\approx 0.3679$ which is surprisingly close to the complement of the golden ratio $1 - \varphi \approx 0.3824$ (Nake 1974; Völz 1988).

In order to fulfill the three desiderata of von Weizsäcker and von Weizsäcker (1972) above, an appropriate framework for music reception had already been proposed by Meyer (1956), that can be straightforwardly applied to aesthetic appreciation in general:

We have stated that styles in music are basically complex systems of probability relationships [...] (Meyer 1956, p. 54)

The listener brings to music not only specifically musical experiences, associations, and dispositions but also important beliefs as to the nature and significance of aesthetic experience in general and the expected musical experience in particular. (Meyer 1956, p. 73)

Revision of opinion, stressed earlier in the discussion of probability, is also important in the perception of form. Here, too, the listener often finds it necessary to revise his opinions of the significance of what has passed and his expectations of what is still to come in the light of an unexpected present. (Meyer 1956, p. 60)

But even more important than designative meaning is what we have called embodied meaning. From this point of view what a musical stimulus or a series of stimuli indicate and point to are not extramusical concepts and objects but other musical events which are about to happen. That is, one musical event (be it a tone, a phrase, or a whole section) has meaning because it points to and makes us expect another musical event. (Meyer 1956, p. 35)

This small selection of quotes motivates to select the theories of *dynamic semantics* (Blutner and beim Graben 2013; Gärdenfors 1988; beim Graben 2006, 2014; Groenendijk and Stokhof 1991; Kracht 2002) and *dynamic update logics* (Baltag and Smets 2008, 2022; van Benthem et al. 2009; Blutner 1996; Veltman 1996) as an appropriate modeling framework for the pragmatic information of aesthetic appraisal. According to Groenendijk and Stokhof (1991, p. 43):

[...] the meaning of a sentence does not lie in its truth conditions, but rather in the way it changes (the representation of) the information of the interpreter. The utterance of a sentence brings us from a certain state of information to another one.

This *dynamic view of meaning* is also supported by Kracht (2002, p. 217):

[...] dynamic semantics is called ‘dynamic’ because it assumes that the meaning of a sentence is not its truth condition but rather its impact on the hearer.

These ideas give rise to the following definitions.

Let $\mathscr {A}$ be a cognitive agent, here regarded as an aesthetic *appreciator* (Nake 1974) with mental belief space $(\mathscr {X}, \textbf{p})$. Thus, a mental state of $\mathscr {A}$ is a probability distribution $\textbf{p}$ over a repertoire $\mathscr {X}$, or briefly, a *belief state* over $\mathscr {X}$ in the sense of either partially observable decision models in artificial intelligence research (Russell and Norvig 2010), or in the sense of Bayesian dynamic update semantics (Baltag and Smets 2008, 2022; van Benthem et al. 2009; Gärdenfors 1988; beim Graben 2006, 2014).

Next, consider a natural or artistic object or event *A*, such as a phrase or a verse of a poem, a view upon a building, a perspective toward a sculpture, a scene in a drama, or a motive or a phrase in music. When *A* can be represented as a mental operator $\textbf{A}$ acting on $(\mathscr {X}, \textbf{p})$ by means of a suitable functor $\Phi : A \mapsto \textbf{A}$, a mapping from an *a priori* belief state $\textbf{q}$ (i.e., a distribution over the repertoire $\mathscr {X}$) to an *a posteriori* belief state $\textbf{p}$ (i.e., another or the same distribution), defined by the action

4 $$\begin{aligned} \textbf{p} = \textbf{A}\, \textbf{q} \end{aligned}$$

of $\textbf{A}$, entails the *dynamic meaning* $\llbracket A \rrbracket$ of the aesthetic object or event *A*, hence

5 $$\begin{aligned} \llbracket A \rrbracket = \textbf{A}: \textbf{q} \mapsto \textbf{p} . \end{aligned}$$

Note that $\textbf{A}$ could be in general a nonlinear mapping from prior to posterior. In case $\textbf{A}$ is a linear operator, it becomes a Markovian stochastic transition matrix as often employed for statistical learning approaches to aesthetic enculturation (Agres et al. 2018; Pearce 2018). Further note that aesthetic operators can be composed by virtue of their action upon priors, i.e.,

6 $$\begin{aligned} \llbracket A; B \rrbracket= &  \llbracket A \rrbracket \circ \llbracket B \rrbracket \end{aligned}$$

7 $$\begin{aligned} (\textbf{A} \circ \textbf{B}) \textbf{q}= &  \textbf{A}(\textbf{B}\, \textbf{q}) . \end{aligned}$$

Here, Eq. (6) expresses that the meaning of the sequence *A*; *B* (with ‘;’ as sequential concatenation) is the composition product of the meanings, $\llbracket A \rrbracket \circ \llbracket B \rrbracket = \textbf{A} \circ \textbf{B}$, defined in (7). The operator product $\textbf{A} \circ \textbf{B}$ is in general not commutative:

8 $$\begin{aligned} (\textbf{A} \circ \textbf{B}) \textbf{q} = \textbf{A}(\textbf{B}\, \textbf{q}) \ne \textbf{B}(\textbf{A}\, \textbf{q}) = (\textbf{B} \circ \textbf{A}) \textbf{q} . \end{aligned}$$

In this framework, novelty (originality) and confirmation (banality) can be rigorously defined (beim Graben 2006). A mental operator $\textbf{A}$ is called *novel* when it cannot be understood or even processed by the agent $\mathscr {A}$. Therefore, it must act as the identity operator upon any belief state,

9 $$\begin{aligned} \textbf{A} \textbf{q} = \textbf{q} ,\quad \text {for all } \textbf{q} . \end{aligned}$$

Likewise, a mental operator $\textbf{B}$ *confirms* a previously processed operator $\textbf{A}$, if

10 $$\begin{aligned} (\textbf{B} \circ \textbf{A}) \textbf{q} = \textbf{A} \textbf{q} ,\quad \text {for all } \textbf{q} . \end{aligned}$$

After these preparations, the *information gain* for a single element $E_i$ of the repertoire $\mathscr {X}$ (Kharkevich 1960) that is provided by the application of the aesthetic operator $\textbf{A}$ upon a belief state $\textbf{q}$, can be expressed as the difference between the information contents of the prior and its updated posterior,

11 $$\begin{aligned} \Delta I(p_i) = I(q_i) - I(p_i) = - \,\textrm{ld}\,q_i - (- \,\textrm{ld}\,p_i) = \,\textrm{ld}\,\frac{p_i}{q_i} . \end{aligned}$$

Averaging $\Delta I(p_i)$ over all events $E_i \in \mathscr {X}$ with respect to the updated distribution $\textbf{p}$ yields the so-called *Kullback–Leibler divergence* as the average information gain (Kullback and Leibler 1951) between the distributions $\textbf{p}, \textbf{q}$,

12 $$\begin{aligned} D(\textbf{p} \Vert \textbf{q}) = \sum _i p_i \Delta I(p_i) = \sum _i p_i \,\textrm{ld}\,\frac{p_i}{q_i} . \end{aligned}$$

Under the special constraint that $\textbf{A}$ satisfies every desideratum of von Weizsäcker and von Weizsäcker (1972), listed above, the Kullback–Leibler divergence

13 $$\begin{aligned} S(\textbf{p} \Vert \textbf{q}) = D(\textbf{A} \textbf{q} \Vert \textbf{q}) \end{aligned}$$

might be called *pragmatic information* of the aesthetic operator $\textbf{A}$ in belief state $\textbf{q}$ for the dynamic updating (4): $\textbf{p} = \textbf{A}\, \textbf{q}$ (beim Graben 2006). Hence:

- (*i*) Pragmatic information *S* assesses the impact of an object or event *A* upon a mental belief state $\textbf{q}$ of a cognitive agent through its dynamic meaning $\llbracket A \rrbracket = \textbf{A}$, i.e. $\textbf{p} = \textbf{A} \textbf{q}$ (5).
- (*ii*) Pragmatic information *S* vanishes in the limits of novelty (originality) *N* and confirmation (banality) *C*. In the first limiting case (9), it holds $D(\textbf{A} \textbf{q} \Vert \textbf{q}) = D(\textbf{q} \Vert \textbf{q})= 0$, whereas in second one (10), $D((\textbf{B} \circ \textbf{A}) \textbf{q} \Vert \textbf{A} \textbf{q}) = D(\textbf{A} \textbf{q} \Vert \textbf{A} \textbf{q}) = 0$.
- (*iii*) Pragmatic information *S* requires a non-classical but rather quantum-like description in terms of incompatible, i.e. non-commuting mental operators, $\textbf{A} \circ \textbf{B} \ne \textbf{B} \circ \textbf{A}$ (8).

Note that Sun et al. (2011) and Agres et al. (2018) refer to Kullback–Leibler divergence as to predictive information in their own statistical learning approaches, whereas Skyrms (2010) used Kullback–Leibler divergence for the assessment of signals in pragmatic signaling games. Moreover, variational free energy can be expressed as the Kullback–Leibler divergence $D(q(\vartheta ) \Vert p(\tilde{y}, \theta )))$ of the “ensemble density” q(ϑ) given the “generative density” $p(\tilde{y}, \theta )$ (Friston et al. 2006, Eq. (1)), from which predictive processing and active inference are deducible under suitable Bayesian conditionalizations. Under the additional assumption that the ensemble density equals the canonical distribution of statistical mechanics with the logarithm of the generative density as eigenenergy function, their Kullback–Leibler divergence approaches the well-known Helmholtz free energy of equilibrium thermodynamics as a metaphorical justification (Friston et al. 2006, Eq. (1)).

## Modeling aesthetic appraisal

As already mentioned in the Introduction, the present study aims at a particular application of the phenomenological model for aesthetic pragmatic information, outlined in the previous section, to the experimental findings of Cheung et al. (2019). In their first experiment, 39 healthy adults had to listen to 1,039 chords in 30 chord progressions that were selected from 745 pop songs listed in the US Billboard “‘Hot 100” chart between 1958 and 1991, after removing melody and rhythm and after transposition to C major mode. Subjects were instructed to continuously rate the pleasantness of each chord using a mechanical slider during stimulus exposure. To obtain the independent variables, an unsupervised statistical-learning model (Pearce 2018) was trained to assess the statistical regularities of over 80,000 chord progressions from the same music corpus. For each chord presented in the rating experiment, its uncertainty *H* was estimated as conditional Shannon entropy of the model. Similarly, the surprise *I* was determined for every chord through the model. Figure 2a shows the results of Cheung et al. (2019). It displays standardized pleasure ratings P(I‖H), namely the aesthetic appreciation of music, in dependence of two variables: context uncertainty, *H*(*q*), and stimulus surprise, *I*(*p*).

In order to develop a mathematical model for the data plotted in Fig. 2a, one has to ascertain functional relationships between the experimental variables of Cheung et al. (2019). For the sake of simplicity, one considers a binary repertoire $\mathscr {X} = \{ E, F \}$ without any statistical correlations between successive events. Then, the prior $\textbf{q} = (q, 1 - q)$ as well as the posterior $\textbf{p} = (p, 1 - p)$ are parameterized by only one real number each: q∈[0,1] for the *a priori* distribution, and p∈[0,1] for the dynamically updated *a posteriori* distribution, with $\textbf{p} = \textbf{A}\, \textbf{q}$ under the aesthetic operator $\textbf{A}$.

Under those assumptions, the Shannon entropy (2) of the prior,

14 $$\begin{aligned} H(q) = - q \,\textrm{ld}\,q - (1 - q) \,\textrm{ld}\,(1 - q) , \end{aligned}$$

which is known as Fermi–Dirac entropy in this particular form in statistical physics for it is maximized by the Fermi–Dirac distribution in the respective variational calculus (Kapur 1970; Borwein et al. 2011), becomes a suitable measure of context uncertainty for the study of Cheung et al. (2019). Figure 1 displays the Fermi–Dirac entropy as a blue curve.

By contrast, the informativity (1)

15 $$\begin{aligned} I(p) = - \,\textrm{ld}\,p \end{aligned}$$

may be appropriately attributed to the surprise of the aesthetically closing stimulus $\textbf{A}$ in the experiment of Cheung et al. (2019). Finally, the Kullback–Leibler divergence (12), interpreted as pragmatic information (13),

16 $$\begin{aligned} S(p \Vert q) = p \,\textrm{ld}\,\frac{p}{q} + (1 - p) \,\textrm{ld}\,\frac{1 - p}{1 - q} \end{aligned}$$

should be correlated with aesthetic enjoyment P(I‖H) (Cheung et al. 2019) for all possible combinations of p,q∈[0,1].

To further proceed, one has to substitute all occurrences of *p*, *q* in (16) by *H*(*q*) and *I*(*p*), inverting the functions (14) and (15), respectively. For the latter, this is straightforwardly achieved through

17 $$\begin{aligned} p = 2^{-I(p)} . \end{aligned}$$

However, the Fermi–Dirac entropy (14) can be firstly approximated by the logistic map

18 $$\begin{aligned} L(q) = 4 q (1 - q) , \end{aligned}$$

plotted as the red curve in Fig. 1 above. The logistic map is well-known from dynamical system theory (Eckmann and Ruelle 1985). Its inverse function is easily obtained through two branches

19 $$\begin{aligned} q_\pm = \frac{1}{2} \left( 1 \pm \sqrt{1 - L(q)}\right) \end{aligned}$$

with a lower branch $q_-$ and an upper branch $q_+$ by solving the quadratic equation (18).

Inserting (19) and (17) into (16), after exploiting the approximation H(q)≈L(q), yields the expression

20 $$\begin{aligned} S(I(p) \Vert H(q))= &  2^{-I(p)} \,\textrm{ld}\,\frac{2^{1 - I(p)}}{1 \pm \sqrt{1 - H(q)}} \nonumber \\ &  \quad + (1 - 2^{-I(p)}) \,\textrm{ld}\,\frac{2 - 2^{ 1 - I(p)}}{1 \mp \sqrt{1 - H(q)}} \end{aligned}$$

as a phenomenological model for aesthetic appraisal. Note that the function S(I(p)‖H(q)) is divergent for H→0, such that it is not well-defined along the ordinate axis.

## Results

The results of the phenomenological account for pragmatic information of aesthetic appraisal are shown in Fig. 2. Here, Fig. 2a, reproduced from Cheung et al. (2019) with permission of Elsevier, depicts the standardized aesthetic pleasure measure P(I‖H) gathered in the musical rating experiment of Cheung et al. (2019) on the one hand. The color coding reflects the emotional intensity of the subjects’ pleasure rating, ranging from P(I‖H)=-4 (dark blue) to P(I‖H)=+4 (dark red). The independent variables are context uncertainty *H*(*q*) of the *a priori* belief state $\textbf{q}$ plotted along the abscissa, and stimulus surprise (ordinate), measured as informativity *I*(*p*) of the *a posteriori* belief state $\textbf{p}$ under aesthetic dynamic updating, $\textbf{p} = \textbf{A} \textbf{q}$, for the dynamic meaning of a chord $\textbf{A} = \llbracket A \rrbracket$.

On the other hand, Fig. 2b presents the Kullback–Leibler divergence (20) computed for the upper branch $q_+$ of the Fermi–Dirac entropy *H*(*q*) (14) plotted as a model of context uncertainty along the abscissa. The ordinate depicts the informativity *I*(*p*) of the *a posteriori* belief state $\textbf{p}$ modeled through (15). Again, large positive values are indicated by bright red, while very small values are represented by dark blue, here. Because of the divergence of S(I(p)‖H(q)) for H→0, only an interval [ε,1] is shown for the abscissa with small cut-off ε=0.05. The ordinate is plotted for [0, 0.5].

Obviously, there is a remarkable qualitative correlation between the experimental rating data in Fig. 2a and the phenomenologically modeled pragmatic information S(I(p)‖H(q)) in Fig. 2b. Pragmatic information is high for quite predictable stimuli (low *I*(*p*)) in very uncertain contexts (high *H*(*q*)), corresponding to region (I) in Fig. 2a, and also for highly surprising stimuli (high *I*(*p*)) in rather certain contexts (low *H*(*q*)), represented by region (II) in Fig. 2a, thereby reflecting the aesthetic appeal of the cadential closures $\textbf{A}$ in Western tonal music. By contrast, aesthetic pleasure and also pragmatic information are low for expected stimuli (low *I*(*p*)) appearing in certain contexts (low *H*(*q*)), characterizing boredom and banality, as well as for surprising stimuli (high *I*(*p*)) in very uncertain contexts (high *H*(*q*)), reflecting randomness or originality (Moles 1966; Nake 1974, 2012). This finding confirms the second desideratum of von Weizsäcker and von Weizsäcker (1972). However, both quantities differ in the respect, that the experimental data exhibit a saddle in parameter space which is not present for pragmatic information, due to its non-negativity constraint.

**Fig. 3:**
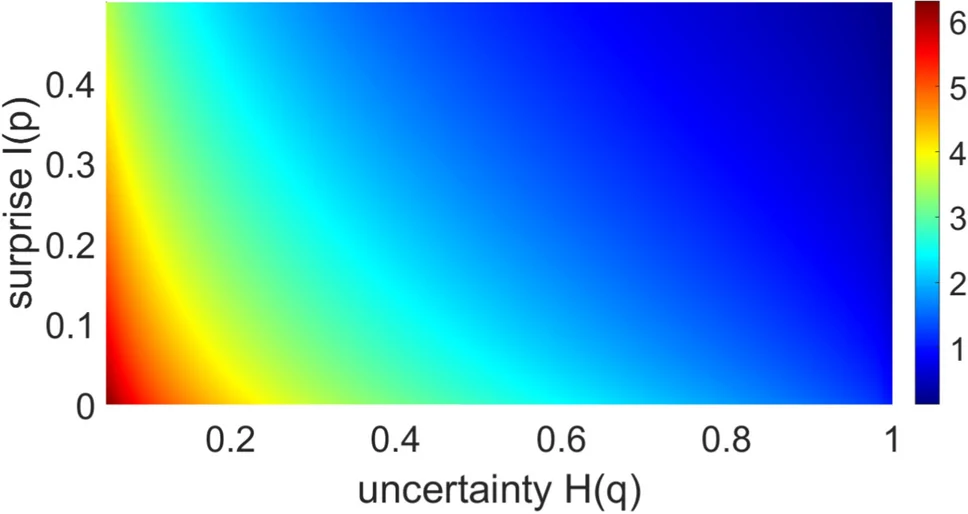
Kullback–Leibler divergence for the lower branch of the probabilistic context updating dynamics (4), depending on context uncertainty *H*(*q*) (abscissa) and stimulus surprise *I*(*p*) (ordinate)

For the sake of completeness, Fig. 3 shows additionally the Kullback–Leibler divergence (20) computed for the lower branch $q_-$ of the Fermi–Dirac entropy (14).

Interestingly, this picture cannot consistently be interpreted as pragmatic information because of the prevailing divergence of S(I(p)‖H(q)) for H→0 that is even present for the interval cut-off ε=0.05 used in Fig. 2b. This behavior indicates that Kullback–Leibler divergence for the lower Fermi–Dirac branch is large for boring stimuli appearing in banal contexts, thus violating the second desideratum of von Weizsäcker and von Weizsäcker (1972) for pragmatic information. Note the huge differences in the scale, depicted by the color bar in comparison with Fig. 2b. Apparently, the divergence $S(I(p) \Vert H(q)) \rightarrow \infty$ for H→0 predominates any other distinction that is induced by the symmetry transformation $q_+ \leftrightarrow q_-$. Therefore, the lower branch of the Kullback–Leibler divergence cannot serve as a viable correlate of aesthetic appreciation.

## Discussion

In their original proposal toward a theory of pragmatic information, von Weizsäcker and von Weizsäcker (1972) formulated three desiderata that could be naturally fulfilled in the framework of dynamic update semantics (beim Graben 2006). First, pragmatic information ascertains the impact of a message or event upon the mental state space of a cognitive agent. In dynamic semantics and machine learning, mental states of a cognitive agent are usually modeled as belief states, namely probability distributions over cognitive repertoires that become updated by novel evidence provided by the reception of messages or events (van Benthem et al. 2009; Blutner 1996; Blutner and beim Graben 2013; Gärdenfors 1988; beim Graben 2006, 2014; Groenendijk and Stokhof 1991; Kracht 2002; Veltman 1996). Second, pragmatic information vanishes in the limits of novelty and confirmation. In dynamic semantics a message or event exhibits novelty if it cannot be understood or even processed by a cognitive agent, whereas confirmation can be expressed by means of logical consequence. Computing the Kullback–Leibler divergence for both special kinds of dynamic updating entails naught. Thus, beim Graben (2006) suggested this quantity as a suitable measure of pragmatic information. Third, pragmatic information requires a quantum-like treatment of incompatible operators. In dynamic semantics, belief revision, default reasoning, and anaphora present paradigmatic examples of non-commutative and hence incompatible updating processes (beim Graben 2006, 2014). Moreover, a description of belief states in terms of Hilbert space vectors leads straightforwardly to a quantum-like formalism (Baltag and Smets 2008, 2022; Huber-Liebl et al. 2022). Related approaches of pragmatic information have been used by Atmanspacher and Scheingraber (1990), Gernert (2006) and, more recently, by Davis et al. (2024).

The present study has demonstrated that the framework of pragmatic information in dynamic semantics can be immediately applied to aesthetics as the science of beauty (Baumgarten 1750). Under the assumption that aesthetic objects or events act as quantum-like operators upon the belief states of a cognitive agent (Meyer 1956), called the *appreciator* in the present context (Nake 1974), hedonic value is minimized in cases of plain banality and overburdening originality (Moles 1966; Nake 1974, 2012; Völz 1988). Applied to a recent study on the appreciation of closure effects (cadences) in Western tonal music (Cheung et al. 2019), the suggested measure of pragmatic information provides a phenomenological model of the experimental results. Vanishing in the cases of banality and originality, pragmatic information qualitatively correlates with high aesthetic pleasure for predictable closures in rather uncertain contexts and also for surprising closures in more certain contexts.

The unpretentious phenomenological model of aesthetic appraisal presented here, is based on a binary repertoire $\mathscr {X} \equiv \mathscr {X}_0$ of maximal coarse grain that produces a stochastic Bernoulli process of uncorrelated events. In order to discuss computational models of aesthetic appreciation as well, one has to consider a hierarchy of repertoires $\mathscr {X}_k$ ($k \in \mathbb {N}_0$), where $\mathscr {X}_{k + 1}$ is a refinement of $\mathscr {X}_k$. Nake (1974, 2012) described aesthetic appreciation as an iterative algorithm that converges toward some optimal $\mathscr {X}_n$ where the Shannon entropy of the repertoire $\mathscr {X}_n$ has dropped below a given memory capacity, while that of its immediate refinement $\mathscr {X}_{n+1}$ slightly exceeds that threshold.

A related, though quite different account, was suggested by Mizraji (2023). He illustrated the effect of aesthetic emotions by means of photomosaics (Silvers 1996), where a repertoire of a certain grain $\mathscr {X}_n$ is provided by the mosaic tiles comprising some picture on a much larger scale. beim Graben (2024) has further augmented Mizraji’s (2023) argument by drawing an analogy to the *Beauty of Fractals* (Peitgen and Richter 1986). This analogy allows for an interpretation of Kant’s philosophical aesthetics, mentioned in the Introduction (Kant 1790/1914). Identifying “Imagination” in Kant’s theory with the capability to select appropriate repertoires and his “Understanding” with the faculty of data compression of an aesthetic object or event, the “free harmonious play” of the mental faculties describes a never-ending, though converging process of aesthetic valuation, $\lim _{n \rightarrow \infty }\mathscr {X}_n$.

This interpretation is further supported by the neural reinforcement learning account suggested by Schmidhuber (2010) and Goodfellow et al. (2014). As demonstrated by beim Graben (2024), Kant’s “Imagination” can be identified with the creative generator module and “Understanding” with the categorizing discriminator module of a generative adversarial neural network (GAN) (Goodfellow et al. 2014; beim Graben 2024).

The GAN model also provides a neurodynamical explanation of the experimental data from Cheung et al. (2019). In the neural network, Kant’s “free harmonious play” is represented by an adversarial game between the generator module and the discriminator module, where the generator attempts to hocus the discriminator and vice versa (beim Graben 2024). Then, high aesthetic pleasure for predictable closures in rather uncertain contexts (region I) reflects reward for the discriminator beating the generator, while high aesthetic pleasure for surprising closures in almost certain contexts (region II) indicates reward for the generator, this time beating the discriminator. This is consistent with phenomenological pragmatic information.

Moreover, the algorithm’s convergence could be related to criticality in neurodynamical systems (Cessac et al. 2004; Haimovici et al. 2013; Papo and Buldú 2023; Stam and de Bruin 2004). In the subcritical case, the algorithm would terminate after a finite number of steps, indicating banality and boredom, while in the supercritical case, the algorithm would be divergent, not approaching any convergent regime. Only for criticality, convergence of the algorithm could be achieved, thereby reflecting the appreciation of beauty.

In order to relate the present model to the predictive processing framework (Frascaroli et al. 2024; Friston et al. 2006; Huron 2006; Koelsch et al. 2019; de Cruys et al. 2024; Constant et al. 2024; Kesner 2024; Leder and Pelowski 2024; Miller et al. 2024; Pepperell 2024), one has to consider a variant thereof, namely epistemic affordance theory (Koelsch et al. 2019; Huron 2019), which distinguishes between content prediction (of the first kind) and meta-prediction (of the second kind). Huron’s ITPTRA theory (Huron 2006), attributing positive emotional reward to successful content prediction in rather certain contexts, seems to be at variance with the experimental findings of Cheung et al. (2019) and their neurodynamical modeling accounts, described above. However, unpredictability in certain contexts (region II) affords model revision through active inference, while predictable unpredictability (of the second kind) in uncertain contexts (region I) leads to positively valenced surprise for successful predictions (Constant et al. 2024; Huron 2019; Koelsch et al. 2019).

Finally, some future directions of the present approach may be indicated. First of all, scholars from empirical aesthetics are invited to test the suggested phenomenal model against their experimental data by means of statistical modeling and correlation analyses (Agres et al. 2017; Cheung et al. 2019, 2024; You et al. 2023). Second, the unpretentious approximation of the Dirac-Fermi entropy (14) by the logistic map (18) might be improved by solving (14) in terms of the Lambert *W*-function (Borwein and Lindstrom 2016). Third, instead of the simple two-parameter Bernoulli process, more sophisticated stochastic dynamical systems, such as (hidden) Markov processes (Agres et al. 2018; Pearce 2018), Bayesian neurodynamics (Sun et al. 2011), or reinforcement learning architectures (such as GAN) may be explored (Schmidhuber 2010; Goodfellow et al. 2014). The latter seems particularly appropriate for that aim, as the GAN objective function could also be expressed in terms of Kullback–Leibler divergence, and hence pragmatic information.
